# Brighter Time: A Smartphone App Recording Cognitive Task Performance and Illuminance in Everyday Life

**DOI:** 10.3390/clockssleep4040045

**Published:** 2022-10-21

**Authors:** Marina Gardasevic, Altug Didikoglu, Samuel J. D. Lawrence, Céline Vetter, Timothy M. Brown, Annette E. Allen, Robert J. Lucas

**Affiliations:** 1 Centre for Biological Timing, Division of Neuroscience and Experimental Psychology, School of Biological Sciences, Faculty of Biology Medicine and Health, University of Manchester, Manchester M13 9PT, UK; 2 Department of Integrative Physiology, University of Colorado Boulder, Boulder, CO 80309, USA; 3 Centre for Biological Timing, Division of Diabetes Endocrinology and Gastroenterology, School of Medical Sciences, Faculty of Biology Medicine and Health, University of Manchester, Manchester M13 9PT, UK

**Keywords:** light exposure, memory, attention, alertness, visual search, cognitive performance, smartphone app

## Abstract

Light is an influential regulator of behavioural and physiological state in mammals. Features of cognitive performance such as memory, vigilance and alertness can be altered by bright light exposure under laboratory and field conditions. However, the importance of light as a regulator of performance in everyday life is hard to assess and has so far remained largely unclear. We set out to address this uncertainty by developing a tool to capture measures of cognitive performance and light exposure, at scale, and during everyday life. To this end, we generated an app (Brighter Time) which incorporated a psychomotor vigilance (PVT), an N-back and a visual search task with questionnaire-based assessments of demographic characteristics, general health, chronotype and sleep. The app also measured illuminance during task completion using the smartphone’s intrinsic light meter. We undertook a pilot feasibility study of Brighter Time based on 91-week-long acquisition phases within a convenience sample (recruited by local advertisements and word of mouth) running Brighter Time on their own smartphones over two study phases in winter and summer. Study compliance was suitable (median = 20/21 requested task completions per subject). Statistically significant associations were observed between subjective sleepiness and performance in all tasks. Significant daily variations in PVT and visual search performance were also observed. Higher illuminance was associated with reduced reaction time and lower inverse efficiency score in the visual search. Brighter Time thus represents a viable option for large-scale collection of cognitive task data in everyday life, and is able to reveal associations between task performance and sleepiness, time of day and current illuminance. Brighter Time’s utility could be extended to exploring associations with longer-term patterns of light exposure and/or other light metrics by integrating with wearable light meters.

## 1. Introduction

The natural daily rhythm in ambient light is reflected in substantial 24 h variations in many aspects of human behaviour and physiology. This association between ambient light and physiology can be accounted for in part by synchronisation of the circadian clock with the light:dark cycle, and in part by more direct effects of bright light on physiological and behavioural state [1,2]. Among the parameters under this dual circadian and photic control are determinants of cognitive performance, such as alertness and reaction time [3].

There is a growing understanding of the neurophysiological mechanisms linking light exposure to aspects of cognitive performance (see, e.g., [4,5,6,7,8]). Moreover, associations between brighter light exposure and improved alertness and/or cognitive performance have been reported in controlled laboratory and field experiments [9,10,11,12,13,14,15,16,17,18,19,20,21]. However, not all studies have observed an impact of light on performance (see, e.g., [21,22,23,24,25,26]), and there is evidence that effects may be quite context-specific (e.g., [21,27,28]). The true significance of natural variations in light exposure as a determinant of performance in real-world settings thus remains uncertain. One approach to address this deficit would be to determine how cognitive performance correlates with light exposure in natural populations outside of experimental conditions. Collecting such data would reveal the circumstances under which naturally occurring variations in light exposure within and between individuals have a significant influence on cognitive performance. Population-level studies of this type require two types of technology: a meter capable of logging each subject’s personal light exposure, and a method for incorporating objective task-based measures of cognitive performance into everyday life.

The goal of this study was to establish the feasibility of a smartphone-based approach to the problem of collecting measures of cognitive performance and light exposure in everyday life. Smartphones have intrinsic light meters whose output is employed in determining optimal screen brightness and camera settings and can provide a measure of illuminance [29]. At the same time, we reasoned that adapting a selection of cognitive tasks for presentation using a smartphone app could allow these tasks to be easily incorporated into everyday life. In this way, we aimed to develop a methodology to allow cognitive tasks to be performed at any time and under almost all circumstances, while simultaneously measuring light exposure.

## 2. Methods

### 2.1. Recruitment and Procedure

Seventy volunteers were recruited via local advertisements and word-of-mouth. These participants passed the following recruitment exclusion criteria: current sleep disorders, eye disorders resulting in visual impairment, current consumption of medication known to affect sleep, and recent (within 2 weeks) travel across time zones. All participants were at least 18 years of age and based in the United Kingdom. The study was run at 2 times of the year: January to March 2021 and July to August 2021. The former coincided with a strict COVID-19 lockdown in the U.K., during which people were told to stay home except for outdoor exercise and essential activities; educational and leisure establishments were closed, as were non-essential shops; people could socialise outdoors in groups of up to 6; and a face mask mandate was in place (https://commonslibrary.parliament.uk/research-briefings/cbp-9068/ accessed on 10 October 2022). A less strict lockdown (socialising indoors in groups of up to 6 allowed; non-essential businesses open; mask mandate in place) was in place for part of phase 2 (until 19 July 2021). Participants were asked to play games at least 3 times per day, preferably in the morning, middle of the day and evening. In phase 1, 65 volunteers completed the study, and 68 volunteers completed phase 2. Amongst these, 21 of the volunteers completed both phases. This project was carried out with the ethical approval of the University of Manchester Research Ethics Committee (Ref: 2020-8667-12901). Users provided informed consent through the app. Participants installed and used the app on their own Android smartphone (with participants self-declaring that the screen was not cracked or damaged).

For all tasks, reaction times less than 100 ms were defined as errors and discarded. Entries from subjects with less than 40% correct answers for that task amongst all trials were also discarded (1.2% of all entries were discarded because of insufficient accuracy). If all ambient light measurements were zero for a given participant, light sensor readings were considered erroneous and were nullified (3 out of 91 individual observations). Participants who completed a task at least 8 times (independent of how many days they played) were included in the analysis. For PVT, 22.5% of participants failed this criterion, leaving 69 participants in the final analysis; for NB, 29.5% of participants failed, leaving 31 participants (note that only N-back data from phase 2 were eligible for inclusion); and for VS, 24.4% of participants failed, leaving 65 participants.

### 2.2. Software and Hardware

**Figure 1:**
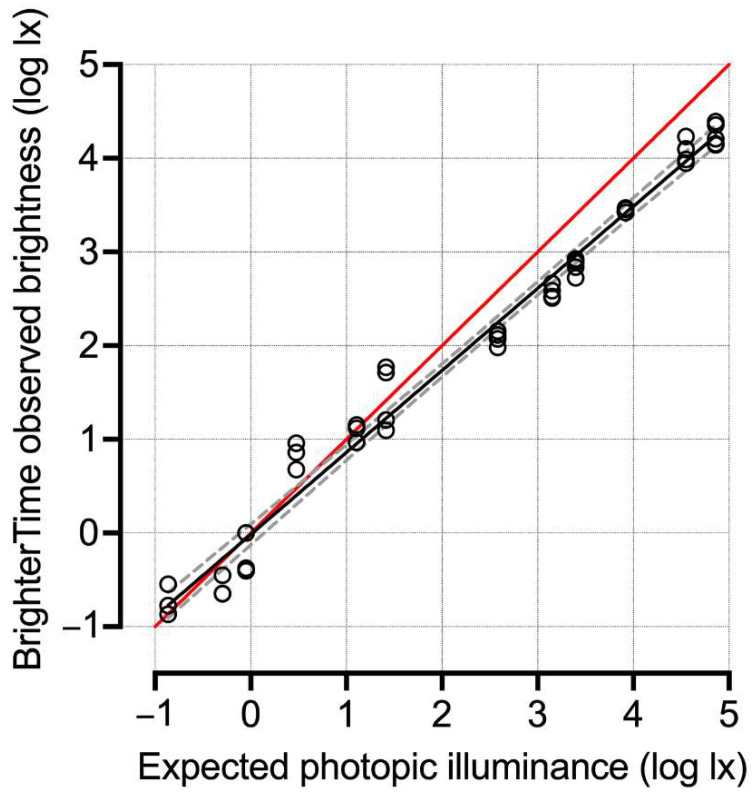
Comparison of Brighter Time light measures with calibrated standards. Illuminance was measured by the Brighter Time app using an Android smartphone (Samsung M51) under exposure to a range of calibrated (SpectroCAL MKII Spectroradiometer, Cambridge Research Systems) illuminances (from 0.1 lx to 10,000 lx) produced by a yellow LED light in a dark box. Black circles show each observation. Expected and observed light measurements were compared using linear regression (black line shows the regression fit line and dashed grey lines show 95% confidence interval). The red line shows the ideal fit line (slope = 1).

The Brighter Time app was created in Xamarin.Forms 4.8 by Dr. Adrian Harwood of ResearchIT Manchester. The game engine was built using SkiaSharp 2.80.2 graphics API and game pages are implemented as a Xamarin. The form content pages are presented with a SkiaSharp canvas embedded edge to edge. The games were conceptualised and created in Unity (V2018.3.3f1). The collected data were stored in the University of Manchester ResearchIT’s Storage Connect service. The app was designed to be used on Android phones because access to the sensors is restricted on iOS. Ambient light levels (lux) were captured during gameplay using the phone’s forward-facing ambient light sensor. Mean illuminance was reported as reading from the light sensor for the duration of the task (sampled every 60 ms). To assess the efficacy of the Brighter Time light measures, a test Android smartphone (Samsung M51) running the app was exposed to a range of calibrated illuminances (from 0.1 lx to 10^5^ lx; SpectroCAL MKII Spectroradiometer, Cambridge Research Systems, Rochester, United Kingdom) produced by a yellow LED light in a dark box. Expected and measured illuminances were highly correlated (Figure 1), but the app slightly underestimated illuminance at the brighter settings (linear regression slope = 0.88; 95%CI = 0.8393 to 0.9120; Pearson R^2^ = 0.98; *p* < 0.001).

### 2.3. Tasks

**Figure 2:**
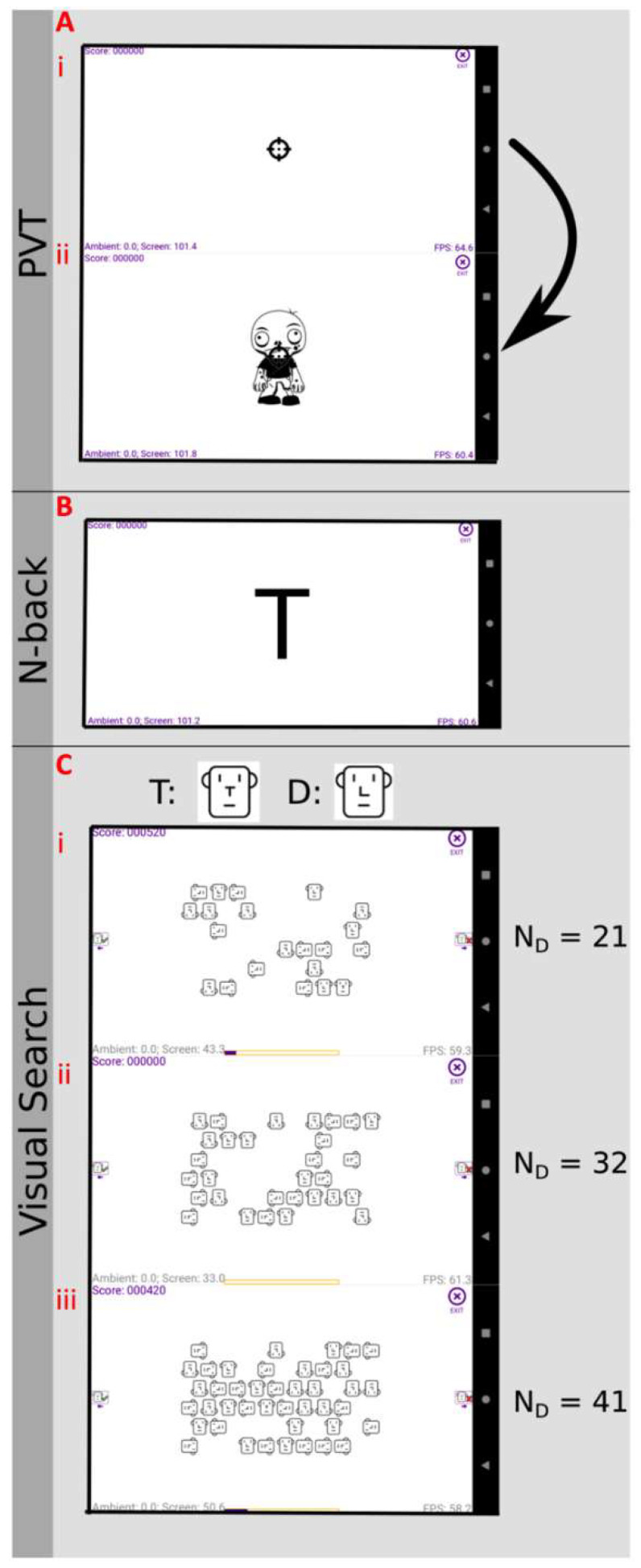
Screenshots of the cognitive tasks used in Brighter Time. (**A**) In the PVT task, participants viewed a screen with a central fixation cross (**i**) and were asked to touch the screen when a zombie (**ii**) appeared. (**B**) The N-back memory task comprised sequentially presented letters, with the participant required to touch the screen when the letter presented was the same as one 2 presentations prior. (**C**) The visual search task required participants to determine whether a ‘monkey’ target image was present against a field of ‘man’ distractors. Screenshots for either (**i**) 21, (**ii**) 32 or (**iii**) 41 distractors. ‘Monkey’ and ‘man’ images were identical except the nose was T- or L-shaped, respectively.

For our app, we chose three tasks: a psychomotor vigilance task (PVT) to measure sustained attention; an N-back task to measure working memory; and a T vs. L visual search task to measure search accuracy and efficiency (Figure 2). All tasks were presented using monochrome images (apart from correct/incorrect indication in the N-back). We produced ‘game’ versions of the PVT and visual search tasks with the aim of improving participant engagement and take up. As reaction time is a key performance metric for these tests, we undertook simulations to confirm that measuring this parameter was in principle compatible with smartphone sampling rates (Supplementary Figure S1).

The visual PVT was chosen due to its ease of administration and near absence of a learning curve [30]. The game version of PVT was ‘Zombie Shooting’ (Figure 2A), whereby participants fixated on a crosshairs target and were vigilant for a zombie appearing in its place. When they tapped the screen, this was translated into ‘shooting’ the zombie, and one of four simple headshot animations played. The cross occupied a space of 64 × 64 dp and the zombie was 208 dp. The user was required to respond as quickly as possible, and their reaction time and accuracy were recorded. A single session consisted of 37 trials with a 2–10 s inter-stimulus interval randomly selected for each presentation. The stimulus time-out was 1 s. The total test duration was approximately 5 min.

To assess working memory, we chose the N-back task (Figure 2B; [31]). A 3-back version of the task was presented in phase 1, in which participants were sequentially presented with letters sampled from A, B, D, E, K, M, R, S and T and required to indicate, by pressing anywhere on the screen, when the letter presented was the same as the one 3 presentations prior (e.g., A-E-S-A-K). A green bar appeared at the bottom of the screen if they were correct and a red bar if their selection was false or if they missed an N-back target. The font was Anonymous Pro with size 256 dp. Stimuli were presented for a maximum of 2 s, and the inter-stimulus interval was 1 s. Each trial consisted of 15 targets amongst >300 trials. For each trial, the reaction time and accuracy were recorded. Participants complained about how long it took to complete this version of the task (>6 min) and the infrequent appearance of targets. In addition, task accuracy was very low in some subjects, indicating that they did not understand the task. For phase 2, we refined the N-back to improve compliance, switching to a 2-back version (e.g., A-B-A-D-T) and presenting 15 targets among 45 trials, parameters used in other studies [14,32]. Only the phase 2 data were included in the analysis for the N-back task.

The visual search task we selected was a gamified version of T vs. L. In this task, participants played ‘Find the Monkey’ in which, they searched for a ‘monkey’ as the target among ‘men’ as distractors (Figure 2C). The man and monkey icons were identical, bar the nose, which was a ‘T’ for monkey and an ‘L’ for man. The distractors could take one of four (0°, 90°, 180°, 270°) orientations while the target is 0°-oriented. The participant was asked to indicate as quickly as possible if the target (monkey) was present or absent in the scene. In the app, this is achieved by tapping the left half of the screen for ‘target present’ and the right half for ‘target absent’. The participants were immediately informed if their response was correct or incorrect and, in the case of the target being present, its location was revealed. Each trial consisted of 120 presentations with a 50/50 split for present/absent trials and equal amounts of three distractor numbers of 21, 32 and 41. The time-out for response was 10 s, with an inter-stimulus interval of 2 s. For each trial, the reaction time, accuracy and the number of distractors were recorded. Given that density and character size are important determinants in search efficiency, we chose to have the visual search arena a constant size (400 × 240 dp) across phones, with the arena on larger phones being surrounded by a white border identical to the background, and the characters a constant size of 30 × 32 dp [16,33].

Tasks were shown always in the same sequence: PVT, N-back, visual search. Data were saved after each task, and in rare instances (7% of sessions), not all tasks were completed. All tasks presented the participants with running scores; these were subtle and in the corner of the screen for the duration of the task and were centred on the screen upon task completion. Correct responses increased the score, with more points for faster reaction times, while misses or false responses reduced the score. The lower end of the score was capped at zero to prevent negative motivation. Participants were given immediate feedback as to whether they missed (time out) or were correct or chose an incorrect option (in the case of N-back and visual search).

### 2.4. Surveys and Measures

As part of the feasibility assessment for the Brighter Time app, we asked users to create a profile by completing introductory questionnaires (Supplementary Table S1) capturing the type of information that could in principle be relevant for stratifying data in larger datasets. This comprised questions relating to general demographic factors; sleep, eye, psychological and neurological disorders; recent (2 weeks) travel across time zones; average weekly caffeine and alcohol consumption; and smoking habits. They indicated if they were breast feeding, had young children (<1 years of age) in their household and any medication they were taking. They were asked their subjective chronotype. Chronotype was also assessed with the Munich Chronotype Questionnaire (MCTQ) [34]. General activity levels were assessed with the International Physical Activity Questionnaire (IPAQ) [35]. Only upon completing these questionnaires and providing consent were the tasks available to the participants.

Participants were asked to complete all three tasks (PVT, N-back and visual search) multiple times a day for the period of 1 week. In the registration, participants practiced all games before starting the study. Upon each opening of the app, participants were prompted to report their sleepiness with a modified (10 point) version of the Karolinska Sleepiness Scale (KSS) [36], their caffeine and alcohol consumption for that day, their sleep and wake times for the previous evening, and any naps they have taken (Supplementary Table S2). Whenever a task was initiated, participants were also required to indicate how long they had been in their current lighting conditions.

### 2.5. Statistical Analysis

**Figure 3:**
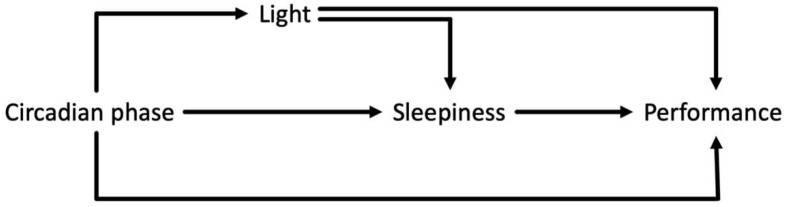
Directed acyclic graph representing hypothesised relationships between light, circadian phase and performance. Light and circadian phase influence performance directly and/or via effects on sleepiness. Circadian phase in turn influences light exposure thanks to the tendency for sleep and rest to occur at low light levels.

All data manipulation, analysis and plotting were conducted in RStudio version 4.0.4 (2021). Our analysis was built around a simple model of how light and circadian phase could influence real-world cognitive performance based on relationships established under laboratory conditions (Figure 3). According to the model, the main predictor variables in our analysis were sleepiness score (1–10 scale, 10 being extremely sleepy), light exposure (log photopic illuminance) and time awake (duration between awakening time and test time). We also separately looked for daily variations in task performance.

In the absence of foreground knowledge about the performance of cognitive tasks as administered in real life using Brighter Time, we structured our analysis to remain agnostic regarding the most appropriate single outcome measure for each task. We therefore included multiple measures, with appropriate correction for multiple comparisons. For all tasks, we analysed hit rate (%correct answers/total presentations) and false alarm rate (%incorrect attempts/total presentations), and median reaction times of correct answers. Additionally, as participants may employ different tactics with regards to speed–accuracy trade-offs, we included Inverse Efficiency Score (IES; average reaction time/proportion correct answers) [37]. For PVT, the number of lapses (slower reaction times than 500 ms) was recorded. For the N-back test, the discriminability index (d′) was calculated to measure individuals’ ability to detect the correct signal. For visual search analysis, we included a measure of search efficiency, calculated as the slope for the reaction time against varying distractor size (ms/item) [38]. Efficient searches have a search time independent of the number of distractors and as such are characterised by a search slope of ≈ 0 ms/item. In tasks where the target closely resembles the distractors (known as ‘difficult searches’), searches are never fully efficient and as such have a slope >0 ms/item. For our visual search analyses, we reported performance outcomes for both target-present and target-absent trials together.

**Figure 4:**
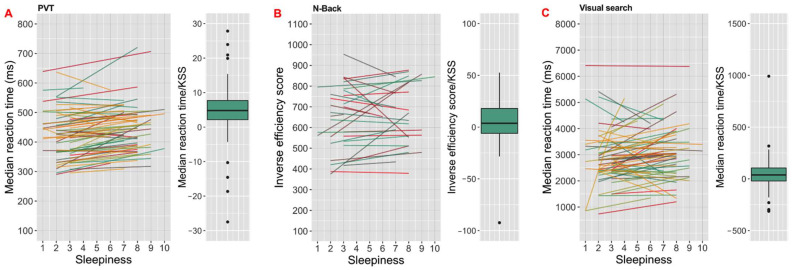
Associations between subjective sleepiness and (**A**) psychomotor vigilance task (PVT) reaction times (ms; median for all presentations in an iteration of the task), (**B**) N-back task inverse efficiency score (ratio of mean reaction time and proportion of correct answers), (**C**) visual search median reaction time (ms). Linear regression fits between KSS score (higher scores are more sleepy) and (**A**,**C)** median reaction times or (**B**) inverse efficiency slopes for each participant record, with the distribution of slopes for fit lines shown in boxplots (box shows median ± inter-quartile range (IQR), whiskers extend to 1.5 × IQR with outliers as closed circles) to the right.

For all analyses, linear mixed models (LMM) were used. Models were computed using the lme4 package in R [39] and lmerTest package in R [40]. Random intercept-only models were created for each outcome variable, with participant as a random effect. For each cognitive task type, five outputs were tested; therefore, the *p*-value significance threshold was accepted as 0.01. We assessed three separate models for each output. The first models aimed to determine Brighter Time’s ability to reveal the expected association between sleepiness and cognitive task performance (Figure 4) and included only KSS score as a predictor. To assess associations with light and circadian phase, the second models included time awake (hour) and ambient light (log lx) as fixed effects. We finally assessed the ability of our approach to reveal daily variations in performance using unimodal trigonometric models where sine and cosine of time of day (radians) were added as predictors in the analyses.

## 3. Results

**Figure 5:**
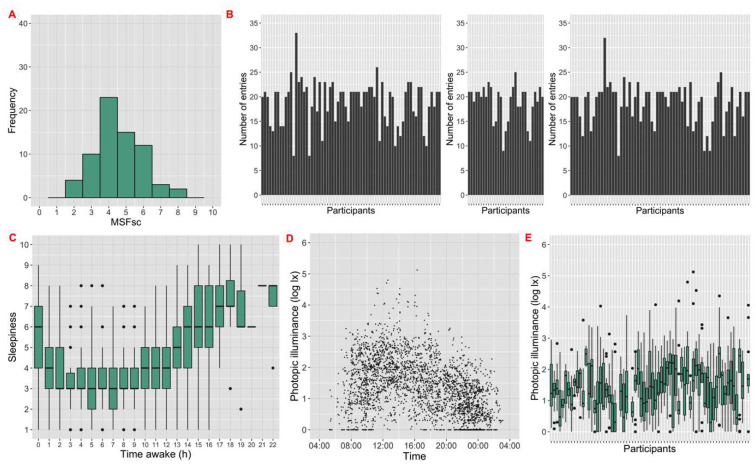
General characteristics of the study sample. (**A**) Histogram of mid-sleep time on free days corrected for sleep debt on workdays (MSFsc) collected by the Munich Chronotype Questionnaire (MCTQ) at the registration baseline. (**B**) Number of cognitive games played for each participant during their study period. The left graph shows the psychomotor vigilance task (PVT) entries, the middle graph shows the N-back entries and the right graph shows visual search entries. (**C**) Change in sleepiness with time awake (N = 3106). Sleepiness was measured using the Karolinska Sleepiness Scale (KSS), with 1 being ‘extremely alert’ and 10 being ‘extremely sleepy, can’t keep awake’. Time awake shows the duration between awakening time and test time. (**D**) Change in ambient light exposure with time of day (N = 2979). Black dots show illuminance measured by the Brighter Time app and reported as log_10_(lux). (**E**) Ambient light exposure (log_10_ lux) measured during the PVT (N = 1248) for 69 participants (green bars).

After exclusions (see Section 2), the PVT task had 69 participant records with 1305 observations and the visual search task had 65 records with 1215 observations. As the N-back task was changed part way through the study (see Section 2), only data from the second phase were included in the analysis (31 records with 586 entries). The majority of participants (69.6%) were aged 18–30 years. There were similar numbers of male and female (52.2%) participants. Participants had a range of physical activity as determined by the international physical activity questionnaire (high: 36.2%, moderate: 33.3%, low: 30.4%). Chronotypes were determined numerically by the Munich Chronotype Questionnaire (MCTQ); we obtained a broad range of chronotype scores (MSF-sc) of 1.6 am–7.8 am (Figure 5A), and the population exhibited a normal distribution (mean: 4.5 am). In response to the subjects’ own assessment of chronotype, 8.7% defined themselves as ‘Definitely a morning type’, 30.4% ‘Rather more a morning type’, 42.0% ‘Rather more an evening type’ and 18.8% ‘Definitely an evening type’. The majority of participants did not smoke (98.6%) and consumed low levels of alcohol (49.3% never consume) and caffeine (median: 5 units per day). The participants were healthy, with only 5.8% anxiety and 4.3% depression rates. None of them were using sleep medication. During the 1-week study periods, participants reported mean sleep duration of 7.8 h (SD = 1.7). They woke on average at 08:20 am (SD = 1.8 h), and mean sleep onset was at 00:33 am (SD = 1.4 h).

The median number of entries per record was 21 for PVT (maximum = 33) and 20 for N-back and visual search (maxima = 25 and 32, respectively) (Figure 5B). There were entries representing most times of day with median entry time around 15:00. The median time awake at the time of entry was 8.4 h, with a maximum of 22.9 h. The median KSS at the time of task completion was 4. Participants generally reported minimum sleepiness around 4–7 h after their awakening (Figure 5C). Descriptive statistics of performance parameters for all three games are provided in Table 1.

The study had a median ambient light reading of 22 lx and a range of 133,000 lx (Figure 5D); these values are broadly as expected for a device used primarily indoors but also available outdoors, and measuring light in either the vertical or horizontal plane [41,42,43]. Importantly, each participant performed tasks across a range of time of day and light conditions (Figure 5D,E). Across all participants, there were 540 days of light and cognition data. Of these, 51 days had data from at least one game played in bright light (>1000 lx).

**Figure 6:**
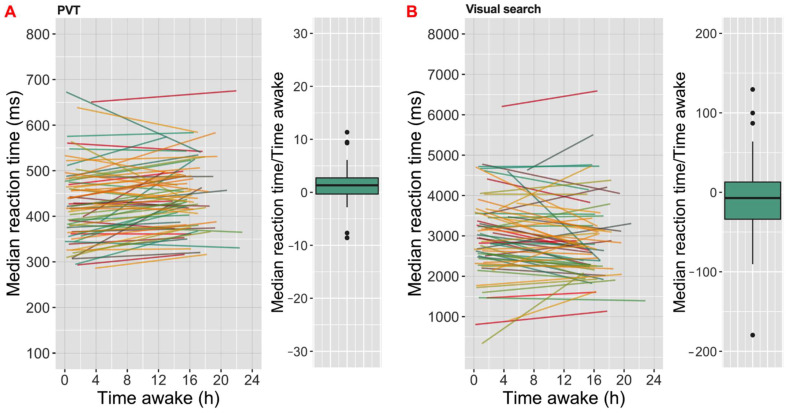
Associations between time awake (h) and (**A**) psychomotor vigilance task (PVT) reaction times (ms; median for all presentations in an iteration of the task), (**B**) visual search median reaction time (ms). Linear regression fits between time awake and (**A**,**B**) median reaction times for each participant record, with the distribution of slopes for fit lines shown in boxplots (box shows median ± inter-quartile range (IQR), whiskers extend to 1.5 × IQR with outliers as closed circles) to the right.

Associations with sleepiness were observed for all three of our tasks. In the case of PVT, statistical analyses revealed negative correlations of sleepiness with performance in attention (Table 2; Figure 4A). Thus, higher sleepiness scores were associated with longer reaction time (coef. = 5.37 ms/KSS; Figure 4A), increased number of lapses (coef. = 0.46 number of lapse/KSS) and higher inverse efficiency score (coef. = 6.99 score/KSS). Consistent with these findings for the PVT results, sleepiness was associated with lower visual search performance (Table 3; Figure 4C and Figure 6B). Higher sleepiness was correlated with higher reaction time (coef. = 47.76 ms/KSS), false alarm rate (coef. = 0.37 %/KSS) and inverse efficiency score (coef. = 66.54 score/KSS), as well as decreased hit rate (coef. = −0.37 %/KSS). We had fewer records for the N-back short-term memory task, because of problems with the way the task had been presented in phase 1 of the study. In this smaller sample size, the only relationship between task performance and predictors was between inverse efficiency score and the KSS (Table 4). Sleepiness increased the score (coef. = 13.13 score/KSS; Figure 4B).

**Figure 7:**
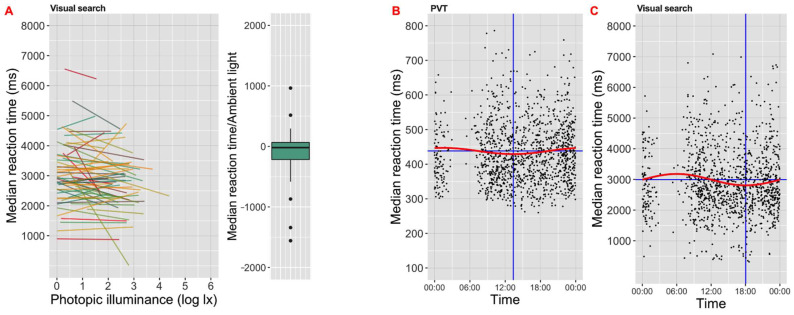
(**A**) Associations between visual search reaction time and illuminance (log lx). Linear regression fits between reaction time (median for all presentations in an iteration of the task) and illuminance for each participant record, with the distribution of slopes for fit lines shown in boxplots (box shows median ± IQR, whiskers extend to 1.5 × IQR with outliers as closed circles) to the right. (**B**,**C**) Associations between time of day and (**B**) psychomotor vigilance task (PVT) reaction times (ms) and (**C**) visual search reaction times (ms). Scatter plots showing the distribution of visual search reaction times across all participant records as a function of time of day. The red lines show the harmonic fit (see Section 2), with horizontal blue line indicating mesor and vertical blue line showing the time of nadir.

Turning to the other elements of our conceptual model (Figure 3), we found associations between task performance and time awake for both PVT and visual search, and between illuminance and performance for visual search (Table 2 and Table 4). For time awake, longer duration since awakening was associated with increases in reaction time (coef. = 1.35 ms/h; Figure 6A) and inverse efficiency score (coef. = 2.25 score/h), and also reduced hit rate (coef. = −0.15%/h) in the PVT. Conversely, longer time awake was associated with shorter reaction time (coef. = −16.71 ms/h; Figure 6B) and higher search efficiency (lower search slope; coef. = −0.57 search slope/h) in the visual search. Higher illuminance was associated with reduced reaction time (coef. = −133.95 ms/log lx; Figure 7A) in the visual search.

The differing effects of time awake on PVT and visual search were also apparent in the time-of-day analysis. There was a significant daily variation in both tasks, but a difference in the time for highest performance. For PVT, median reaction time (Figure 7B), number of lapses and inverse efficiency score all peaked at around 1 p.m. The time-of-day model showed that visual search reaction time was the fastest (Figure 7C) and inverse efficiency score the lowest around 18:00. 

## 4. Discussion

We report the generation of a smartphone app, Brighter Time, capable of simultaneously recording local light intensity and performance in attention, working memory and visual search tasks on a user’s own Android device. Inclusion of a simple questionnaire allows these data to be related to demographic parameters and self-reported sleep. We further show in a pilot feasibility study that Brighter Time is able to reveal associations between subjective sleepiness (KSS) and aspects of performance in all three of the cognitive tasks (PVT, N-back and visual search) under real-world conditions. It also revealed daily variation in PVT and visual search performance, and an association between illuminance and aspects of visual search.

The primary objective of this work was to establish a method for capturing information on light exposure and cognitive task performance at scale and under real-world conditions. Brighter Time has a number of advantages for this purpose. It employs devices (Android smartphones) that are already an intrinsic element of many lives. Smartphones are designed to function across all commonly encountered light intensities and, thanks to their portability, often accompany their user throughout their waking hours. We attempted to make the cognitive tasks more engaging by ‘gamifying’ two of them and to keep the total time taken to complete them as short as possible. In this study, we asked participants to access Brighter Time three times per day, but did not specify times of day to do so, nor was remuneration dependent on the number of tasks completed. Nevertheless, the median number of iterations for each task was ≥20 across 7 days. Given that there were three tasks, it follows that many subjects completed >63 Brighter Time tasks in a week. Recruitment and subject initiation were also efficient. Brighter Time could be made available to download from an app store, and installed and activated without experimenter involvement. The combination of easy accessibility and user engagement raise the possibility of using Brighter Time to collect large amounts of data.

Turning to the quality of cognitive task data produced by Brighter Time, our sustained attention reaction time measures were broadly comparable with lab-based measurements. Thus, in a lab-based sleep deprivation protocol, Grant and colleagues reported reaction times between 250 and 500 ms according to sleepiness level for a 10-minute PVT applied on a computer and between 200 and 300 ms for equivalent data collected on a smartphone for a 3-minute PVT [44]. Our mean reaction time in PVT is 430 ms, which is within this range, but long for a population that was not sleep deprived and for a task run for a relatively short duration (around 5 min) and on a smartphone. This suggests that running the Brighter Time PVT in the real world captures a higher variation in performance than the similar smartphone paradigm achieved in the laboratory conditions employed by Grant et al. [44]. We are less confident about our memory outputs of the N-back because fewer participants completed this. In phase 1 of the study, we employed a three-back version, but this proved too difficult (based on participant feedback and number of records with poor performance), either because of the intrinsic nature of the task under real-world conditions, or because it was imperfectly explained to the participants. We therefore switched to a two-back version for phase 2. However, our results show that the two-back test may have been too easy, with 96% accuracy, which is higher than previous reports [45]. The N-back test thus requires further optimisation (e.g., employing a shorter version of the three-back task) in future versions of Brighter Time to achieve an appropriate level of difficulty. 

One concern with any cognitive task is that performance may improve over time as subjects become more adept at the task and/or develop more effective approaches (learning). Our participants practised all three tasks as part of the study initiation, but we did not include a specific learning phase in the protocol. To determine whether learning was a substantial consideration, we assessed changes in task performance over time (Supplementary Figure S2). Neither the PVT nor N-back tasks showed evidence of a strong learning effect in this analysis. Visual search task reaction times did seem to improve across the study. Accounting for this visual search learning curve in protocol design could be improve future studies.

A potential downside of collecting data from the subject’s own smartphone is that measures of cognitive function could be impacted by variations in performance across devices. We did not undertake a systematic investigation of device-to-device variation in Brighter Time performance, which in any case could never be comprehensive given the huge range of smartphone models in circulation (never mind variation within models). Nevertheless, a feeling for the scale of the problem can be achieved by comparing PVT reaction time as a function of smartphone manufacturer in our dataset (Supplementary Figure S3). Reaction times are of particular concern as they require accurate logging of subject response with respect to the precise timing of stimulus presentation. The plot of reaction time by the manufacturer reveals that, while this parameter was broadly similar, there was not full overlap of inter-quartile ranges across devices. We cannot be sure whether such variability reflects systematic variation in device performance or imperfect distribution of subjects with varying PVT performance across the smartphone models. In either event, this plot confirms the desirability of accounting for inter-individual variability in design and analyses of studies using Brighter Time to avoid introducing bias, and the need to account for the potential for device-to-device variability to increase variance in task performance measures when considering effect sizes and statistical power.

Ultimately, Brighter Time’s utility is defined by its ability to reveal influences on cognitive performance. Figure 3 captures our hypothesised influences on cognitive performance. As sleepiness is a common route via which both light and circadian time could influence performance, we were especially interested in whether Brighter Time was able to reveal associations between sleepiness and aspects of task performance. A positive correlation between sleepiness and reaction time in vigilance tests has been reported under controlled conditions (e.g., [3,46]). Brighter Time confirmed that such an association was also apparent in our sample population during everyday life, with higher KSS scores (greater sleepiness) being associated with longer reaction times, more lapses and higher inverse efficiency in the PVT in our dataset. The impact of sleepiness was also apparent on other tasks, with inverse efficiency score for the N-back task and reaction time, false alarm rate and inverse efficiency on visual search, all indicating poorer performance at higher KSS scores.

The Brighter Time dataset was also able to reveal daily variations in performance for the PVT and visual search. Interestingly, the optimal time of day for these two tasks was different. Highest performance on PVT measures was in the early afternoon, whereas the peak in visual search performance was delayed by around 5 hours to the early evening. This implies that in our sample population, while sustained attention was highest around the middle of the day, at least some of the processes required for visual search peaked in the early evening [47].

Brighter Time provided tentative evidence for an association between illuminance and performance. In our analysis, we accounted for the strong correlation between illuminance and time of day (Figure 5D) by employing a model including both factors. This returned significant associations between time of day and several parameters of the PVT and visual search task. Accounting for this time-of-day effect, an association between illuminance and visual search performance was observed. Higher illuminance was associated with shorter reaction times which is an indicator of improved performance. The magnitude of this effect was comparable with that associated with natural variations in subjective sleepiness, with an estimated 250 ms reduction in vs. reaction time across 10–1000 lx, equivalent to that predicted for a five-point change in KSS. However, interpretation of this outcome should take account of the likely association between illuminance and screen brightness, which itself could impact performance [48]. In designing Brighter Time, we considered disabling the automatic screen brightness adjustment but decided that this would have a greater impact on the association between ambient light level and screen visibility. Changes in screen brightness are an unavoidable consequence of running these tasks under very divergent lighting, and the user’s application of automatic screen brightness adjustment functions (or other methods) represents a reasonable approach to ensure suitable visibility under all circumstances (although under direct sunlight these maybe insufficient).

The Brighter Time approach has a number of limitations when it comes to measuring light exposure. Our own validation and published work [29] indicates that Android light meters provide a reasonable measure of illuminance, unlikely to approach the performance of fully calibrated lux meters, but probably adequate to track large variations in light exposure [41]. However, the smartphone light meter measures only illuminance, rather than other more appropriate metrics such as melanopic irradiance [49,50]. Moreover, it measures light in the direction that the smartphone is pointing, which is, by definition, approximately opposite to the participant’s direction of view. In addition, participants might be using sunglasses, so the observed illuminance may not be the true magnitude an individual was exposed to. Finally, and perhaps most importantly, while Brighter Time can measure illuminance at the time of task performance, there is no easy way for it to provide an accurate log of light history. We know from experimental manipulations that integrated light exposure over up to several hours can impact performance and other non-image forming responses [19,51]. In principle, the app could record the smartphone’s measure of illuminance continuously, but that would have a detrimental effect on battery life, and the smartphone’s exposure to light could be different from that of its user when not in use. In an attempt to address this problem, Brighter Time did request a response to the question ‘How long you had been in your current lighting environment?’ before each task completion. In practice, the appearance of implausible responses (>12 h) indicated that subjects interpreted this question in different ways, and we did not include it in our analysis.

In summary, Brighter Time represents a viable option for collecting cognitive task performance across waking hours over several days in everyday life from naïve subjects without specialist knowledge or training. Its ease of use for both researcher and participant means that it is readily scalable for large dataset collection. Brighter Time can also collect responses to simple questionnaires, meaning that it can be used to explore relationships between performance and demographic parameters, as well as self-assessed behavioural state and sleep logs. While there may be room for improvement in the design of the cognitive tasks (especially N-back), Brighter Time’s biggest limitation for our purposes is its method of light measurement. Integration of the data collection elements of Brighter Time with wearable light loggers represents an exciting opportunity to objectively assess associations between cognitive performance and light exposure in real-world populations.

## Figures and Tables

**Table 1:** Descriptive statistics of cognitive task outcomes.

	Min.	1st Quantile	3rd Quantile	Max.	Median	Mean	Standard Deviation
**PVT**							
Median reaction time (ms)	260.0	366.0	483.0	860.0	419.5	431.3	88.6
Hit rate (%)	40.5	91.9	100.0	100.0	97.3	94.7	7.6
False alarm rate (%)	0.0	0.0	2.7	43.2	0.0	2.1	3.6
Inverse efficiency score	271.8	392.7	555.4	1644.0	456.6	487.2	90.3
Number of lapses	0.0	2.0	14.0	37.0	7.0	9.4	8.6
**N-back**							
Median reaction time (ms)	329.5	454.2	694.8	1192.0	571.0	587.0	161.6
Hit rate (%)	46.7	93.3	100.0	100.0	100.0	95.7	7.7
False alarm rate (%)	0.0	0.0	3.3	26.7	3.3	3.2	4.0
Inverse efficiency score	336.3	511.6	789.8	1876.4	635.9	667.8	213.4
d′	1.0	3.0	4.0	4.0	3.5	3.4	0.6
**Visual search**							
Median reaction time (ms)	315.0	2225.8	3514.5	7084.5	2747.5	2924.6	1094.2
Hit rate (%)	40.2	84.3	94.1	100.0	90.2	87.1	10.6
False alarm rate (%)	0.0	5.9	14.7	59.8	9.8	12.6	10.3
Inverse efficiency score	887.0	2570.0	3969.0	14,069.4	3131.8	3356.6	1198.9
Search efficiency slope (ms/item)	−51.6	19.5	54.0	147.7	36.1	39.0	28.7

The PVT task had 69 participants with 1305 observations. The visual search task had 65 participants with 1215 observations. The N-back task had 31 participants with 586 entries.

**Table 2:** Statistical models of psychomotor vigilance task (PVT) outcomes.

	Median Reaction Time (ms)	Number of Lapses	Hit Rate (%)	False Alarm Rate (%)	Inverse Efficiency Score
**Model-1**					
Intercept	413.13	7.82	94.89	1.90	467.04
KSS					
Coef.	5.37	0.46	−0.16	0.07	6.99
SE	0.77	0.08	0.08	0.05	1.30
*p*	**6.01 × 10^−12^**	**9.08 × 10^−9^**	6.53 × 10^−2^	1.29 × 10^−1^	**9.76 × 10^−8^**
**Model-2**					
Intercept	434.14	9.36	95.96	1.83	482.37
Time awake (h)					
Coef.	1.35	0.11	−0.15	0.05	2.25
SE	0.49	0.05	0.05	0.03	0.83
*p*	**5.69 × 10^−3^**	2.95 × 10^−2^	**6.59 × 10^−3^**	1.26 × 10^−1^	**7.02 × 10^−3^**
Ambient light (log lx)					
Coef.	−4.37	−0.20	−0.65	0.04	−0.49
SE	3.19	0.32	0.35	0.20	5.46
*p*	1.71 × 10^−1^	5.46 × 10^−1^	6.56 × 10^−2^	8.42 × 10^−1^	9.29 × 10^−1^
Time awake × Ambient light					
Coef.	−0.56	−0.06	0.03	0.00	−0.51
SE	0.34	0.03	0.04	0.02	0.57
*p*	9.37 × 10^−2^	9.35 × 10^−2^	4.30 × 10^−1^	9.95 × 10^−1^	3.79 × 10^−1^
**Model-3**					
Mesor	438.18	9.89	94.19	2.26	499.71
Cosine					
Coef.	8.43	0.53	−0.40	0.25	12.87
SE	1.86	0.19	0.20	0.11	3.12
*p*	**6.49 × 10^−6^**	**5.29 × 10^−3^**	4.66 × 10^−2^	2.26 × 10^−2^	**3.98 × 10^−5^**
Sine					
Coef.	3.21	0.06	0.07	0.10	3.91
SE	2.48	0.25	0.27	0.15	4.15
*p*	1.95 × 10^−1^	8.03 × 10^−1^	7.95 × 10^−1^	5.01 × 10^−1^	3.46 × 10^−1^
Amplitude	9.02	0.53	0.41	0.27	13.45
Nadir (clock time)	13:23	12:27	23:20	13:26	13:08

Linear mixed models of PVT outcomes (median reaction time, number of lapses, hit rate, false alarm rate, inverse efficiency score). Three separate models were performed for each outcome. Model-1 predictor: Karolinska Sleepiness Scale (KSS). Model-2 predictor: time awake (h) + ambient light (log lx) + time awake × ambient light. Model-3 predictor: cosine (2π × time of day/24) + sine (2π × time of day/24). Bold results are significant after correction for multiple testing (*p* < 0.01).

**Table 3:** Statistical models of visual search task outcomes.

	Median Reaction Time (ms)	Slope (Reaction time/Number of Distractor)	Hit Rate (%)	False Alarm Rate (%)	Inverse Efficiency Score
**Model-1**					
Intercept	2736.72	36.71	88.16	11.48	3115.93
KSS					
Coef.	47.76	0.60	−0.37	0.37	66.54
SE	10.36	0.36	0.09	0.08	13.16
*p*	**4.44 × 10^−6^**	9.54 × 10^−2^	**1.72 × 10^−5^**	**7.14 × 10^−6^**	**4.91 × 10^−7^**
**Model-2**					
Intercept	3248.42	45.74	87.03	12.60	3708.16
Time awake (h)					
Coef.	−16.71	−0.57	−0.07	0.05	−15.74
SE	6.51	0.22	0.05	0.05	8.34
*p*	**1.04 × 10^−2^**	**1.05 × 10^−2^**	2.15 × 10^−1^	3.04 × 10^−1^	5.95 × 10^−2^
Ambient light (log lx)					
Coef.	−133.95	−2.64	0.05	−0.05	−150.59
SE	46.48	1.59	0.37	0.36	59.57
*p*	**4.03 × 10^−3^**	9.86 × 10^−2^	8.86 × 10^−1^	8.94 × 10^−1^	1.16 × 10^−2^
Time awake × Ambient light					
Coef.	0.05	0.18	0.00	0.01	1.93
SE	4.76	0.16	0.04	0.04	6.10
*p*	9.92 × 10^−1^	2.74 × 10^−1^	9.53 × 10^−1^	7.38 × 10^−1^	7.52 × 10^−1^
**Model-3**					
Mesor	2992.66	39.68	86.47	13.16	3459.68
Cosine					
Coef.	−1.25	−0.84	−0.40	0.38	13.20
SE	23.91	0.84	0.20	0.19	30.62
*p*	9.58 × 10^−1^	3.15 × 10^−1^	4.48 × 10^−2^	4.75 × 10^−2^	6.67 × 10^−1^
Sine					
Coef.	186.21	1.55	−0.17	0.20	202.96
SE	32.34	1.13	0.27	0.26	41.42
*p*	**1.10 × 10^−8^**	1.72 × 10^−1^	5.39 × 10^−1^	4.39 × 10^−1^	**1.10 × 10^−6^**
Amplitude	186.21	1.76	0.43	0.42	203.39
Nadir	18:02	19:54	01:30	13:51	17:45

Linear mixed models of visual search task outcomes (median reaction time, slope (reaction time/number of distractor), hit rate, false alarm rate, inverse efficiency score). Three separate models were performed for each outcome. Model-1 predictor: Karolinska Sleepiness Scale (KSS). Model-2 predictor: time awake (h) + ambient light (log lx) + time awake × ambient light. Model-3 predictor: cosine (2π × time of day/24) + sine (2π × time of day/24). Bold results are significant after correction for multiple testing (*p* < 0.01).

**Table 4:** Statistical models of N-back task outcomes.

	Median Reaction Time (ms)	d′	Hit Rate (%)	False Alarm Rate (%)	Inverse Efficiency Score
**Model-1**					
Intercept	563.86	3.47	97.20	2.91	612.82
KSS					
Coef.	5.62	−0.02	−0.36	0.06	13.13
SE	2.37	0.01	0.16	0.09	3.52
*p*	1.80 × 10^−2^	1.06 × 10^−1^	2.51 × 10^−2^	5.05 × 10^−1^	**2.09 × 10^−4^**
**Model-2**					
Intercept	639.83	3.37	94.53	2.47	744.37
Time awake (h)					
Coef.	−3.16	0.00	0.06	0.02	−3.96
SE	1.29	0.01	0.09	0.05	1.94
*p*	1.44 × 10^−2^	7.71 × 10^−1^	5.25 × 10^−1^	7.00 × 10^−1^	4.22 × 10^−2^
Ambient light (log lx)					
Coef.	−21.63	0.02	0.49	0.22	−29.66
SE	8.69	0.05	0.60	0.32	13.13
*p*	1.32 × 10^−2^	7.39 × 10^−1^	4.12 × 10^−1^	4.87 × 10^−1^	2.43 × 10^−2^
Time awake × Ambient light					
Coef.	1.02	0.00	−0.02	0.03	0.91
SE	0.90	0.00	0.06	0.03	1.36
*p*	2.57 × 10^−1^	4.25 × 10^−1^	7.09 × 10^−1^	4.19 × 10^−1^	5.04 × 10^−1^
**Model-3**					
Mesor	591.74	3.38	95.45	3.12	676.42
Cosine					
Coef.	−4.64	0.00	−0.07	−0.02	−5.69
SE	5.53	0.03	0.38	0.20	8.28
*p*	4.02 × 10^−1^	9.37 × 10^−1^	8.60 × 10^−1^	9.28 × 10^−1^	4.92 × 10^−1^
Sine					
Coef.	11.73	0.01	−0.43	−0.30	17.39
SE	7.05	0.04	0.48	0.26	10.56
*p*	9.69 × 10^−2^	8.24 × 10^−1^	3.73 × 10^−1^	2.52 × 10^−1^	1.00 × 10^−1^
Amplitude	12.61	0.01	0.43	0.30	18.30
Nadir (clock time)	19:26	19:02	05:25	05:46	19:13

Linear mixed models of N-back task outcomes (median reaction time, d′ discriminability score, hit rate, false alarm rate, inverse efficiency score). Three separate models were performed for each outcome. Model-1 predictor: Karolinska Sleepiness Scale (KSS). Model-2 predictor: time awake (h) + ambient light (log lx) + time awake × ambient light. Model-3 predictor: cosine (2π × time of day/24) + sine (2π × time of day/24). Bold results are significant after correction for multiple testing (*p* < 0.01).

## Data Availability

Data are available in an open access repository https://doi.org/10.48420/20212265 (accessed on 1 January 2022).

## Supplementary Materials

The following supporting information can be downloaded at: https://www.mdpi.com/article/10.3390/clockssleep4040045/s1, Table S1: App registration questionnaire; Table S2: Questions upon every log on; Figure S1: Simulated impact of smartphone temporal resolution on reaction times; Figure S2: Learning effects in the PVT, N-back and visual search cognitive tasks; Figure S3: Varying PVT performance across the smartphone makes.
