# Supplementary material for: Brighter Time: A Smartphone App Recording Cognitive Task Performance and Illuminance in Everyday Life
Source: Clocks Sleep. 2022 Oct 21;4(4):577–94. doi: 10.3390/clockssleep4040045 (PMC9589962; doi:10.3390/clockssleep4040045)
Supplement: Supplementary file 1 [file clockssleep-04-00045-s001.zip › clockssleep-1846828-supplementary.pdf]

Brighter Time: A Smartphone App Recording Cognitive Task Performance and Illuminance in Everyday Life.

Supplementary materials

Supplementary Table S1: App registration questionnaire

Questions	Answers
How old are you?	• Under 18<br>• 18 – 25<br>• 26 – 30<br>• 31 – 35<br>• 36 – 40<br>• 41 – 45<br>• 46 – 50<br>• 51 – 55<br>• 56 – 60<br>• 61 – 65<br>• 65 <
What is your sex?	• Male<br>• Female<br>• Other<br>• Prefer not to answer
(if sex = female)<br>Are you currently pregnant or breast feeding?	
You may have heard of “morning” and “evening” types of people. Which of these do you consider yourself to be?	• Definitely a “morning” type<br>• Rather more a “morning” type than an “evening” type<br>• Rather more an “evening” type than a “morning” type<br>• Definitely an “evening” type
Do you frequently smoke cigarettes (more than 1/day)?	
(If yes) Approximately how many cigarettes/day?	• Under 5<br>• 5 – 10<br>• 10 – 20<br>• Over 20
How much of the following caffeinated drinks do you consume in a typical week?	Coffee (excluding decaf) .... cups<br>Tea (excluding decaf) .... cups<br>Energy drinks .... cups
How many units of alcohol do you drink in a typical week? (1 unit = half a pint of beer, a small glass of wine, a shot of spirit)	.... units
Has a healthcare professional ever told you that you are colour blind?<br>(If yes) which?	• Red/green colour blind<br>• Blue/yellow colour blind<br>• Unsure
Has a healthcare professional ever told you that you have a sleep disorder?	• Insomnia<br>• Sleep apnoea<br>• Restless leg syndrome

	• Other (write in)<br>• Unsure
Has a healthcare professional ever told you that you have an autism spectrum disorder (such as autism or Asperger syndrome)?	
Has a healthcare professional ever told you that you have a mental health disorder?<br>(If yes) which?	• Depression<br>• Anxiety<br>• Schizophrenia<br>• Personality disorder<br>• Other (write in)<br>• Unsure
Has a healthcare professional ever told you that you have eye disease?<br>(If yes) Which?	• Glaucoma<br>• Retinal Degeneration<br>• Macular Degeneration<br>• Cataract<br>• Other<br>• Unsure
Has a healthcare professional ever told you that you have a brain/nerve disorder?<br>(If yes) which?	• Stroke<br>• Parkinson’s<br>• Multiple Sclerosis<br>• Other (write in)<br>• Unsure
Do you take any of these medications frequently (at least 1/week)?	• High blood pressure medication (such as beta-blockers)<br>• Nicotine-replacement products (including gum and nicotine-containing e-liquids)<br>• Anti-arrhythmics (for heart rhythm problems)<br>• Asthma inhalers<br>• Anti-depressants/Anti-anxiety medication<br>• Sleeping tablets<br>• Attention Deficit Disorder (ADD)/Attention Deficit Hyperactivity Disorder (ADHD) medication<br>• Thyroid hormones<br>• Oral contraceptives (birth control pills)<br>• Other (please specify)
Have you travelled across time zones in the past 2 weeks?<br>Several trips?	
(If yes) What was the maximum time difference from your home?	X hours<br>Ahead/behind
(If yes) When did you return?	• 1 – 5 days ago<br>• 6 – 10 days ago<br>• 11 – 14 days ago
Have you worked night shifts in the past two weeks?	
Do you have any children in your household under the age of 1?	

Supplementary Table S2: Questions upon every log on

**Questions**	**Answers**
Consider the last 10 minutes, How would you describe your alertness? [KSS – (Åkerstedt & Gillberg, 1990)]	1. Extremely alert<br>2. Very alert<br>3. Alert<br>4. Rather alert<br>5. Neither alert nor sleepy<br>6. Some signs of sleepiness<br>7. Sleepy, but no effort to keep awake<br>8. Sleepy, but with some effort to keep awake<br>9. Very sleepy, great effort to keep awake, fighting sleep<br>10. Extremely sleepy, can't keep awake
Have you slept (excluding naps) since [date and time of last input]?	
(if yes) When did you fall asleep?	
When did you wake up?	
Have you had any naps today?	
How many caffeinated drinks (cups of coffee/tea/cans of energy drink) have you had today?	
How many units of alcohol have you drank in the last 12 hours? (1 unit = half a pint of beer, a small glass of wine, a shot of spirit)	
Your profile has the following information about your habits in the last 2 weeks:<br>[Travel across time zones]<br>[Night shifts]<br>Are these still correct?	
(if no) [Redirect to relevant questions of registration questionnaire]	

Supplementary Figure S1: Simulated impact of smartphone temporal resolution on reaction times

An important design consideration for Brighter Time was whether smartphones, in principle, likely had sufficient temporal resolution to detect biologically meaningful differences in reaction time. In preparation for the study we therefore ran simulations to determine how smartphone sampling rate could impact differences in reaction time in a published dataset ((Burattini et al., 2019), Table 4, Acoustic Vigilance Test, second half). That study was chosen as it reported means and distribution for reaction times and encompassed potential impacts of a lighting manipulation on this aspect of performance. Briefly: the authors varied colour temperature of ambient lighting and assessed reaction time using an acoustic vigilance task whereby participants were presented with short auditory tones of different frequencies and were required to respond when two identical frequencies were presented in succession. For our simulation, we first asked whether a typical smartphone sampling rate (60Hz) impacted detectable differences in reaction time. On the one hand, we generated simulated data in which the standard deviations for the Burattini et al. control vs experimental groups were retained, but the magnitude of the difference in mean reaction time ($\Delta\mu$) was varied. On the other, we retained the ΔRT (13ms) from Burrattini et al. but varied standard deviation. In both cases, we then applied temporal binning (17ms bins) equivalent to 60Hz sampling frequency to the data and compared actual vs measured $\Delta\mu$ ($\Delta\mu_{Raw}$ vs $\Delta\mu_{60Hz}$). This revealed that even very small $\Delta\mu$ values should be detectable at 60Hz sampling (**Figure S1A**), and that although $\Delta\mu_{60Hz}$ was dependent on standard deviation, this was only a factor when standard deviation was unrealistically small (**Figure S1B**).

Given that smartphone temporal resolution can vary, we then wished to determine the minimum sampling rate require to detect differences in this sample population. We tested sampling our data at a variety of frequencies from 0 to 100Hz at 5Hz intervals. We found a sampling rate as low as 20Hz sufficient to accurately report differences in population mean (**Figure S1Cii**). With regards to standard deviation, sampling rates as low as 10Hz were sufficient to reflect the original population (**Figure S1Cii**).

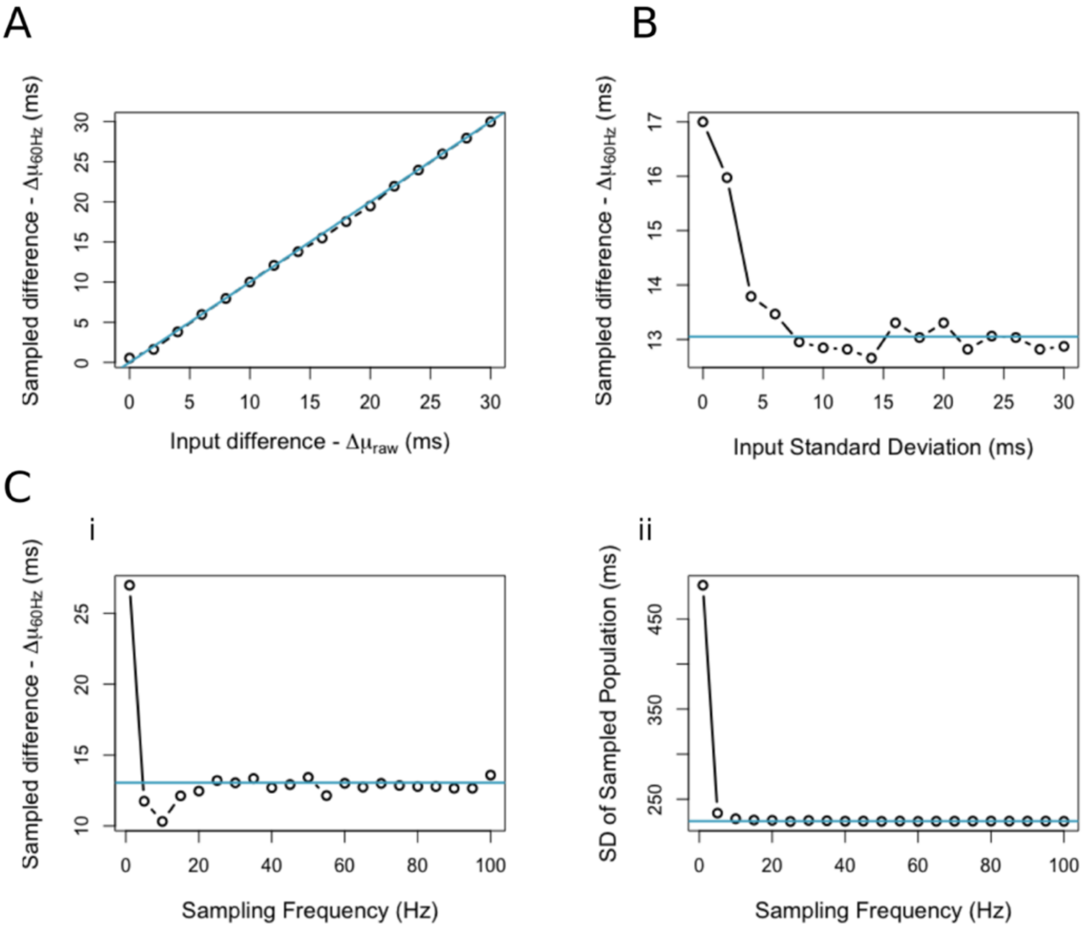

Figure S1. In silico analysis is consistent with smartphone sampling frequencies being sufficient to detect plausible differences in human reaction time. Sampling at 60Hz simulated on published data (Burattini et al., 2019). **A)** Plot showing the difference in mean when sampling at 60Hz ($\Delta\mu_{60Hz}$) for varied differences in mean between the distributions ($\Delta\mu_{raw}$). Line y = x shown in blue. **B)** Plot showing the difference in sampled mean at 60Hz ($\Delta\mu_{60Hz}$) for different input standard deviations. Actual sampled difference 13.05 shown in blue. **C)** Plots showing the effect of varying sampling frequency on **i)** sampled mean ($\Delta\mu_{60Hz}$) and **ii)** sampled standard deviation. Actual population values are shown in blue.

References

Burattini, C.; Piccardi, L.; Curcio, G.; Ferlazzo, F.; Giannin, A.M.; Bisegna, F. Cold LED lighting affects visual but not acoustic vigilance. *Buildi. Environ.* ***2019**, 151*, 148-155.

Supplementary Figure S2: Learning effects in the PVT, N-back and visual search cognitive tasks

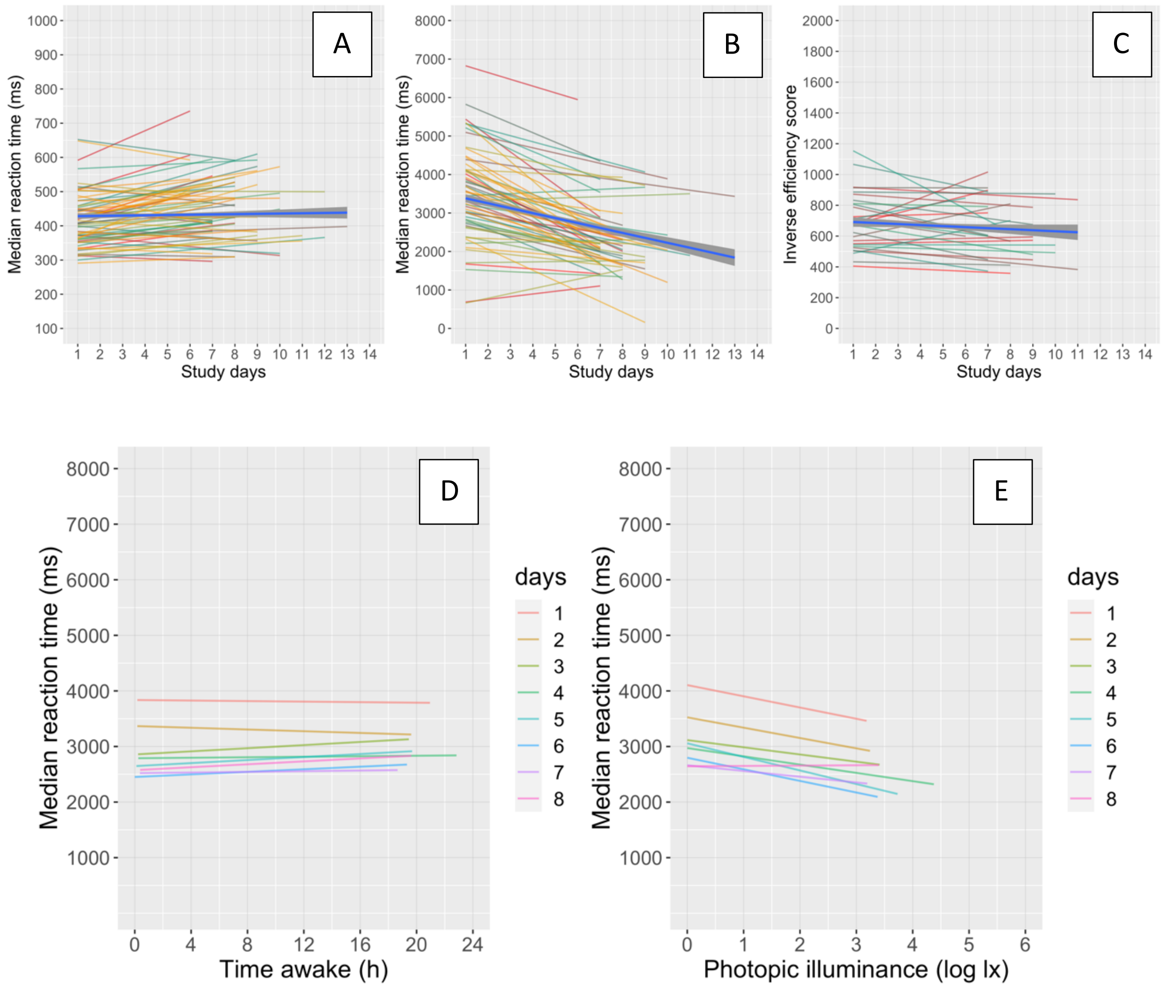

Figure S2. Learning effects in study-specific versions of Psychomotor vigilance task (PVT), N-back task and visual search task. The learning slope of reaction times (ms) in the PVT **(A)** and visual search **(B)**, and inverse efficiency score in the N-back **(C)** are shown. The figures **A-C** show linear regression fits for each participant record, with the blue fit lines showing sample mean fit line and standard error. In the visual search task, reaction times improved significantly across study days (Mixed model with random intercept for participants and random slope for study days: coef.=-160.85 ms, SE=14.58, $p=5*10^{-16}$). The figures **D-E** show linear regression fits for visual search reaction times (ms) as a function of **(D)** time awake (h) and **(E)** illuminance (log lx) on each study day.

Supplementary Figure S3: Varying PVT performance across the smartphone makes

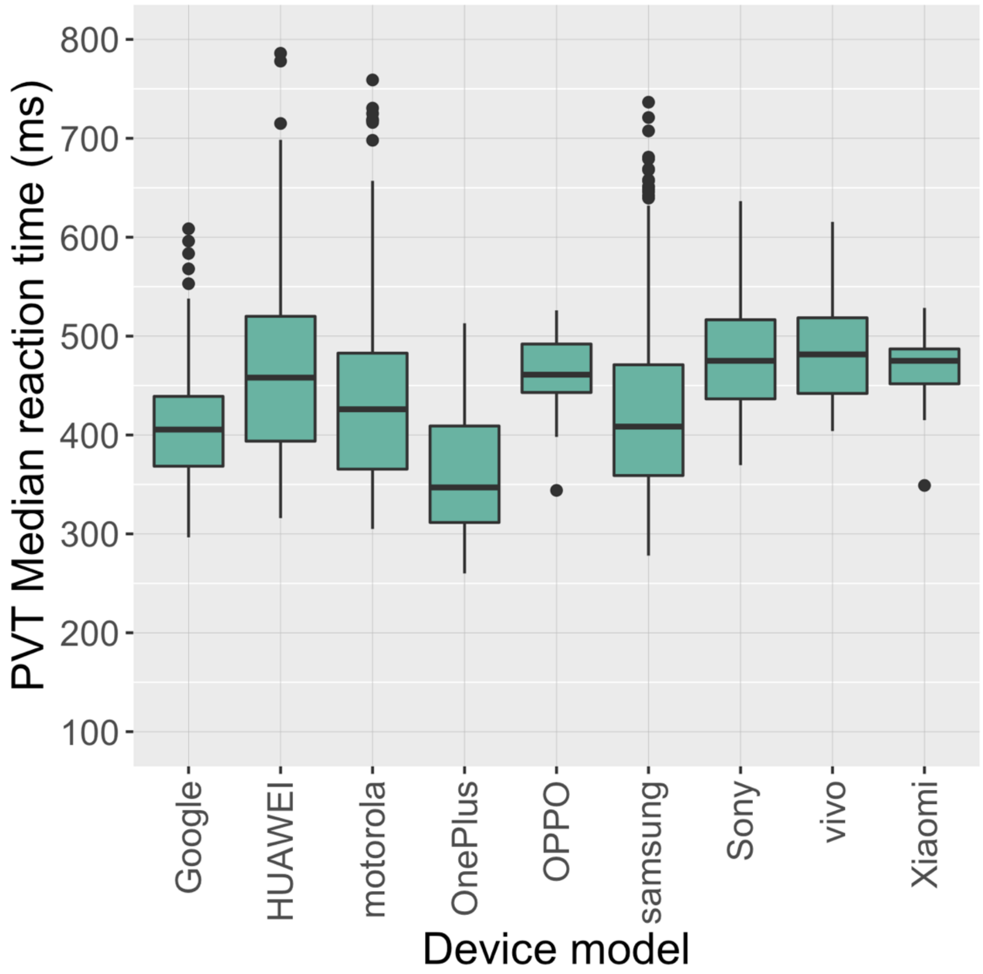

Figure S3. Comparison of Psychomotor vigilance task (PVT) reaction times (ms) collected in this study between different Android smartphone makes. Device make was recorded by the app for every entry for all participants. The figure shows median and interquartile range of reaction times by manufacturer for 69 participants who performed the PVT. While the parameter was broadly similar, there was not full overlap of interquartile ranges across devices. One-way ANOVA resulted in significant medium effect size mean difference between device makes ($F(8,1296)=13.28$; $p<2*10^{-16}$; Eta-squared=0.076).
